# Understanding the marital self-disclosure experience during chemotherapy for gastric cancer: a qualitative study

**DOI:** 10.3389/fpsyg.2025.1572569

**Published:** 2025-05-30

**Authors:** Ye Zhou, Chong Chin Che, Mei Chan Chong, Haiyan Zhao, Yuzhu Hou, Min Zhao

**Affiliations:** ^1^Department of Nursing Science, Faculty of Medicine, Universiti Malaya, Kuala Lumpur, Malaysia; ^2^Editorial Office of International Journal of Nursing Sciences, Chinese Nursing Journals Publishing House Co., Ltd., Beijing, China; ^3^Nursing Department, Jingjiang People’s Hospital Affiliated to Yang zhou University, Taizhou, Jiangsu, China; ^4^Oncology Department, Jingjiang People’s Hospital Affiliated to Yang zhou University, Taizhou, Jiangsu, China; ^5^Department of Gastroenterology, Jingjiang People’s Hospital Affiliated to Yang zhou University, Taizhou, Jiangsu, China

**Keywords:** couple, marital self-disclosure, gastric cancer, qualitative research, spouse

## Abstract

**Objective:**

This study explores the experiences of Chinese gastric cancer patients and their spouses in implementing marital self-disclosure and examines the barriers and facilitators to the acceptance of marital self-disclosure among gastric cancer patients. The study aims to assist nurses in developing effective communication strategies for patients and their spouses, thus promoting their psychological well-being.

**Methods:**

The study employed purposive sampling with a maximum variation approach to select participants. From January to June 2024, 18 pairs of gastric cancer patients and their spouse caregivers, who were hospitalized in the oncology department of a tertiary hospital in Jingjiang, Jiangsu Province, participated in semi-structured face-to-face interviews. Data was transcribed within 24 h after each interview, supported by field notes. Directed content analysis was applied to qualitative content analysis.

**Results:**

Traditional content analysis revealed five overarching themes and 15 sub-themes derived from the experiences of gastric cancer patients and their spouses during chemotherapy. The five themes identified were: (1) perception of marital self-disclosure, (2) the Influence of marital self-disclosure on Couples’ Well-Being, (3) addressing concerns about marital self-disclosure, (4) factors facilitating marital self-disclosure, and (5) the obstacles to marital self-disclosure.

**Conclusion:**

Nurses should recognize the importance of communication between spouses during chemotherapy for gastric cancer patients. Positive interventions should be adopted to alleviate one-sided, conflictual communication and avoidance behaviors. Nurses should focus on personalized communication issues, identify barriers to disclosure promptly, and provide tailored care. Furthermore, patient case management should be emphasized to facilitate the development of more effective communication strategies.

## 1 Introduction

Gastric cancer ranks fifth in the global cancer incidence spectrum and fourth in the death cause spectrum. In 2020, there were 1,089,103 new cases of gastric cancer and 768,793 deaths worldwide, accounting for 5.6 and 7.7% of the total incidence and deaths, respectively (Akbari et al., 2022). The standardized morbidity and mortality rates were 11.1/100,000 and 7.7/100,000, respectively, and the cumulative morbidity and mortality risks of 0–74 years old were 1.31 and 0.90%, respectively. The overall incidence and mortality of non-cardia gastric cancers have declined steadily worldwide for nearly half a century. However, some studies have found that the incidence of gastric cancer (including cardia and non-cardia cancer) in people under the age of 50 has increased in both high-risk and low-risk countries (Arnold et al., 2020). According to the latest cancer statistics released by the National Cancer Registry (Xia et al., 2022), gastric cancer ranks second in cancer incidence and mortality in China, with a total of 679,000 cases and 498,000 deaths, following lung cancer (733,000 deaths).

Gastric cancer has the characteristics of insidious onset, easy missed diagnosis, easy metastasis and high recurrence rate (Akbari et al., 2022). Chemotherapy is the main treatment method for patients with gastric cancer, which can prolong the survival rate of patients with gastric cancer (Sicheng, 2020). Although the progress of medical technology and resection rate for advanced gastric cancer has increased, the high recurrence rate and metastasis rate, as a negative life event, have a great psychological impact on patients (Zhao et al., 2013). Patients with gastric cancer during chemotherapy usually need long-term nutritional support, and chemotherapy patients were suggested to stay at home for a long time during the chemotherapy interval, which means a decrease in income and an increase in disease expenses, which brings a heavy financial burden to the family (Dong et al., 2016), further aggravates the psychological burden, which causes the incidence of fear of cancer recurrence among gastric cancer is high.

The results of multiple linear regression analysis showed that the patient’s self-disclosure and intimacy were the factors that influenced the patient’s fear of cancer recurrence (Fan, 2021). To date, most intervention studies focused on the impact of self-disclosure on the patient’s psychological aspect, ignoring the role of their spouse on the patient’s psychological state (Rabin, 2020; Rafii et al., 2022; Zhang et al., 2021), and failed to treat the patient and their spouse as a whole (Dandan et al., 2018). The unmet supportive needs of cancer patients can lead to lower treatment compliance. These effects can persist for months or even years after treatment, and in severe cases can lead to anxiety disorders, post-traumatic stress symptoms, and depression (Porter et al., 2017).

Theorists proposed an explicitly interactional view of stress and coping processes in the couple context that emphasized partners’ interdependent processes (Bodenmann, 1997). Within the interactional view of stress and coping, partners’ stress is conceptualized as being reciprocal: the stress experiences of both partners are interrelated because one partner’s stress becomes the other partner’s stress (Bodenmann, 1997). Marital dyadic coping is a multidimensional construct that describes both partners’ efforts to deal with stress in the context of the couple’s relationship. These efforts involve what each partner does in terms of communicating stress and providing support to each other, as well as partners’ conjoint efforts to cope with stress.

Marital psychoeducation may help partners learn the value of communicating with one another, providing emotion- and problem-focused support, taking over their spouse’s responsibilities and tasks when their spouse is stressed, and engaging in conjoint efforts to cope when they are both stressed or when one partner is suffering from a severe health condition (Kayser et al., 2007). Improvement of dyadic coping was more favorable for increasing relationship satisfaction than improvement of communication skills in the long run (Bodenmann et al., 2009). In short, dyadic coping is strongly associated with relationship satisfaction. Therefore, incorporating dyadic coping in relationship enhancement programs could be beneficial for the couple’s relationship (Falconier et al., 2015).

Marital self-disclosure refers to the sincere sharing of one’s thoughts and emotions with others through language and written expression, which is a window for individuals to express their emotions, ideas, and opinions to their spouse. The construct of emotional disclosure has been operationalized as both (a) the degree to which individuals express their thoughts and feelings and (b) the degree to which they hold back from doing so (Porter et al., 2009). Emotional disclosure is a central component of the marital emotional support that partners provide to each other (e.g., love, concern, and understanding) (Porter et al., 2009). For patients with cancer who are married or in an intimate relationship, the relationship with their spouse plays a critical role in adapting to their illness. Northouse et al. (2000) suggested that some couples may be at risk of relationship distress as a result of the burdens imposed by the cancer experience. One factor that may lead to deterioration in cancer patient-partner relationships is the challenge of communicating effectively about cancer-related concerns. Married patients with cancer tend to rate their spouses as their most important confidant. However, patients with cancer often feel constrained in talking about their concerns with their spouse, and partners often withdraw or distance themselves from the patient’s emotional distress (Northouse et al., 2000). These avoidant patterns are present even in satisfying couple relationships. Patients’ inability to talk openly with their spouses about their cancer-related concerns may ultimately compromise the quality of the patient-partner relationship as well as the patient’s psychological adjustment. Couples discussing cancer-related concerns may help partners to have a better understanding of patient’s needs and to provide patients with more effective support. In addition, the partner may benefit from hearing the patients talk openly about their concerns because partner depression increases when the patient is worried but not expressing distress; when the patient shares his/her concerns, the partner may experience this as a relief (Porter et al., 2009).

Currently, studies on spousal disease communication have predominantly focused on recoveries from breast, lung, and prostate cancers (Zhang et al., 2019; Çömez and Karayurt, 2020; Nie Zhihong et al., 2019). The treatment regimen for advanced gastric cancer is prolonged, with a high recurrence rate, significantly increasing patients’ psychological burden (Wang et al., 2018). During chemotherapy, gastric cancer patients face dietary restrictions and potential complications such as nausea, vomiting, heartburn, and acid reflux, leading to decreased appetite (Qiuju, 2022). There is a lack of research on spousal communication among gastric cancer patients, and even less on the barriers and facilitators that patients and their spouses encounter when undergoing marital self-disclosure. This research gap provides important insights for designing and conducting marital self-disclosure more effectively. Our team has previously developed an intervention program for marital self-disclosure tailored to gastric cancer patients and their spouses. This study, based on the implementation of marital self-disclosure, conducted qualitative interviews to explore the experiences of Chinese gastric cancer patients and their spouses in the process of marital self-disclosure.

## 2 Methods

The qualitative study adopts a descriptive qualitative research approach, which is a research method based on the philosophical foundation of naturalistic inquiry. It employs strategies such as sampling, data collection, and data analysis, using everyday language and low inferential interpretation to describe the qualitative research of experiences, events, or processes (Colorafi and Evans, 2016). This method follows the principles of natural inquiry to understand the complex experiences within human contexts. When the research question pertains to people’s reactions, thoughts, or factors that promote or hinder certain matters, choosing descriptive qualitative research as a research method is deemed more appropriate (Lambert and Lambert, 2012).

### 2.1 Participants and recruitment

The sample of the qualitative study consists of patients with gastric cancer and their spouses who have participated in marital self-disclosure. The aim is to explore marital self-disclosure experiences. The qualitative used a purposive sampling method for the researchers to select specific participants who experienced the phenomenon being studied as they can provide the best information and obtain an in-depth understanding of the complex experiences of the participants, ensuring diversity in characteristics such as gender, age, residential environment, medical burden, work status, and prior gastric surgery (Grove et al., 2012; LoBiondo-Wood and Haber, 2017). The participants comprised patients diagnosed with gastric cancer and their spouses who were admitted to the oncology department of a tertiary hospital in Hospital A.

The sample size of the qualitative study is determined by data saturation, and no new information emerges from the data collection process except for redundancy from the previously collected data (Grove et al., 2012; LoBiondo-Wood and Haber, 2017; Polit and Beck, 2014). After collecting data from 16 cases, no new information emerged. Two additional interviews were conducted to further verify data saturation. As no new themes surfaced, the sampling process concluded. These additional two cases also met the study’s criteria, bringing the total to 18 couples for analysis. Each interview included two interviewers and the couple, a total of 4 people.

#### 2.1.1 Inclusion criteria of the patient

1. Diagnosed according to the “Guidelines for Diagnosis and Treatment of Gastric Cancer” (Department of Medical Affairs, 2019). Preoperative gastroscopy and pathological diagnosis must confirm advanced gastric cancer.
2. Married and living with their spouse.
3. Males aged ≥22 years and females aged ≥20 years.
4. Possess normal language and cognitive functions and willingly consent to participate.

#### 2.1.2 Inclusion criteria of spouse

1. Married to the patient.
2. Males aged ≥22 years and females aged ≥20 years.
3. Actively undertakes the primary caregiving responsibility for the patient, dedicating ≥4 h daily to caregiving.
4. Has normal language and cognitive functions and willingly consents to participate.

#### 2.1.3 Exclusion criteria of patient and spouse

1. Previously diagnosed with gastric cancer or any other cancer type before the study, exhibiting severe complications such as gastrointestinal obstruction or perforation.
2. Undergoing or has undergone other psychotherapy with a psychiatrist or psychologist.
3. Exhibits cognitive or mental impairments.
4. Suffers from severe visual, auditory, or speech impairments.
5. Currently participating in other research endeavors.
6. The patient and the spouse do not want to participate in the joint interview.

### 2.2 Development of interview outline

An initial interview outline was crafted based on the relationship intimacy model of couple adaptation to cancer and literature review. After consulting with five specialists in oncology, psychology, and gastrointestinal diseases, two patients with gastric cancer were chosen for preliminary interviews (their data was excluded from the result analysis). Incorporating feedback from these patients and expert advice, the interview outline was refined. Researchers initiated interviews with the prompt, “Could you and your spouse share your experiences concerning marital self-disclosure?” to foster rapport with the participants. The primary interview questions are shown in Table 1.

**Table 1:** Interview guide.

a. What does marital self-disclosure mean to you? How did you feel about the marital self-disclosure?
b. What do you think about the effect of marital self-disclosure?
c. What attributes of marital self-disclosure may have impacted fear of cancer recurrence and dyadic coping ability? Give me some examples.
d. What are the factors that affect the implementation of marital self-disclosure?

### 2.3 In-depth individual interview

An interview is a tool for collecting data in a qualitative study. The interview was defined as a form of communication between 2 or more people with specific purposes and is associated with some agreement on a subject matter. A qualitative study aims to obtain the research relevant information from the interviewee to achieve the research objectives, including description, prediction, or explanation of the phenomenon (Dilshad and Latif, 2013). The advantage of using an interview is to explore the inner feelings and attitudes of the informants, such as emotion, feeling of experiences, sensitive issues, and their privileged insights. The interview technique is immensely useful and valuable as it emphasizes a detailed and holistic description of the situation. Therefore, the qualitative interview method has endeavored to appreciate the worldview of the informants and explore the significance of their experiences (Dilshad and Latif, 2013). This study used an in-depth individual interview to collect the qualitative data.

Before beginning each interview, the researcher clearly explained the background and significance of the study to the participants. Written informed consent was obtained from each one. During the interviews, with explicit permission from the participants, the researcher took detailed notes and audio-recorded the entire conversation. The researchers also closely observed and noted the participants’ nonverbal behaviors, such as facial expressions, body movements, and pauses in speech. One primary researcher led the interviews by asking questions and observing, while another researcher ensured the interviewer maintained a neutral stance, maintaining a benign environment for the interview, such as preventing the spouse from engaging in violent verbal or physical conflicts. After each session, the researchers reflected on the discussions, documenting their thoughts and insights in a reflection diary and memo.

### 2.4 Data collection

Before starting the interview, the researchers conducted a systematic search to review the current qualitative research literature on the communication between couples of cancer patients. They also read a large number of related books on qualitative research, and they studied the “Qualitative Research Methods” course, which enabled them to have a clearer understanding of the definition of research problems and a deeper understanding of qualitative research methods and can interview patients to obtain enough information and reduce prejudice, and analysis the related interview data.

Data was collected through semi-structured, in-depth interviews. To minimize the potential influence of medication on the patient’s psychological state, interviews were conducted after hospitalization but before the infusion of chemotherapy drugs. Additionally, the duration of each interview was limited to 40–60 min to accommodate and ensure the participants’ comfort.

The interviews were archived anonymously. All interviews took place in the department’s quiet and cozy rest room, ensuring patient privacy and minimizing interruptions from unrelated staff. In this study, both interviewers are experienced gastroenterology head nurses (co-chief superintendent nurse, female), are nursing graduates with master’s and undergraduate degrees, and have been working in oncology and gastrointestinal departments for over 15 years. They are familiar with patients with gastric cancer and their spouses and had received psychology-related training and passed the national certification examination for psychological consultants. Participants volunteered for this study, and before the interviews, the researchers built a strong rapport and trusted them. In the process of this research, the interviewers played the dual roles of “insider” and “outsider,” respectively. As “insiders,” the two interviewers are head nurses of the oncology and gastroenterology departments. During their clinical work, they had the opportunity to communicate with patients with gastric cancer and formed a good nurse–patient relationship. As “outsiders,” the two interviewers only performed nursing and management work and rarely discussed issues related to marital self-disclosure with patients to avoid preconceived experiences or impressions affecting the overall analysis results.

The researchers utilized a triangulation method, collecting and analyzing data from participants undergoing different chemotherapy cycles and with diverse personal characteristics. This method bolstered the reliability and validity of the research findings by reinforcing the consistency and trustworthiness of the results through varied perspectives and data sources. When participants veered off-topic, the interviewers skillfully steered their responses back to the interview’s core focus, and then the data recorded by two interviewers was integrated.

### 2.5 The qualitative data analysis

The patient’s and their spouses’ views were converted according to the original text without modification or comment, maintaining the accuracy of the original interview information and achieving the highest degree of restoration of the text. This study employs the traditional content analysis method for data analysis (Hsieh and Shannon, 2005). Traditional content analysis is commonly used to describe a research phenomenon, especially when there is limited existing theory and literature related to the phenomenon. Researchers avoid using preconceived categories and allow categories to emerge from the data to generate new insights from the data (Yunxian, 2017).

The steps of the data analysis method include the following:

#### 2.5.1 Obtaining an overall sense of the textual data

Two researchers repeatedly listen to recordings, combine interview notes and reflection diaries, and immerse themselves in the data to gain an overall sense of the data (Yunxian, 2017). The analysis obtained after the first seven interviews in this study is as follows: patients acknowledge and accept marital self-disclosure, understand that marital self-disclosure is beneficial for improving fear of cancer and the relationship between couples and psychological adaptation, are willing to conduct marital self-disclosure according to the nurse’s instruction, and there are still some factors that promote or hinder the implementation of marital self-disclosure.

#### 2.5.2 Open coding

After obtaining an overall sense of the textual data, important ideas and concepts in the data are annotated, and open coding is conducted. Coding is the process of associating keywords or phrases with text fragments, and it is the process of breaking down the data. Two researchers code line by line and sentence by sentence without missing any important information.

#### 2.5.3 Cocodes were grouped to form themes and subthemes

Themes refer to the process of systematically categorizing and conceptualizing certain viewpoints based on coding. Themes are typically formed based on coding, where similar codes are grouped to form themes and sub-themes. Using mind maps, relationships between themes are demonstrated. This process of categorizing themes and sub-themes is iterative. The addition of subsequent interview data may disrupt earlier inductive themes, requiring re-decomposition or the addition of new themes.

#### 2.5.4 Defining themes, sub-themes

After initially obtaining themes and sub-themes, the two researchers continue to compare each interview data, engage in repeated discussions with experts and study team members, and conduct multiple comprehensive summaries. This cycle continues until saturation is reached, meaning no new themes emerge. The final themes are defined, and relevant excerpts are identified from the data.

During the data analysis phase, the researchers maintained an objective stance, momentarily setting aside personal beliefs to circumvent biases that could emerge if their values and emotions influenced the analysis. When the transcribed data was unclear, it was subsequently inquired with the participants for validation. For example, in one interview, a participant used the term “Be Ze Shen,” which translates to “silent” in the local dialect. After transcription, another researcher contacted the participant to verify the translation’s accuracy.

### 2.6 Reliability and validity

Credibility refers to the credibility of the data or belief in the result from the participants’ perspectives. In this study, the researcher used two techniques to improve its credibility. Firstly, the researchers are the oncology department’s psychological counselors and oncology nurses. They are involved in the treatment and nursing care of the patients and are familiar with each other. This mechanism of prolonged engagement was vitally necessary to produce credible findings (Guba, 1985). This process helped the researcher to create trust and a relationship with the participants. Polit and Beck (2014) argued that building rapport and trust with the participants through long-term involvement increases the probability of receiving rich and valuable information from them. The participants became familiar with the researcher by following and caring for the patients. Therefore, they were not surprised when the researcher invited them for an interview.

Secondly, the researcher used peers to debrief to build credibility. Peer debriefing aims to inform the researcher on the state and method of his or her posture and process as well as to maintain valid information in data collection (Guba, 1985). In this study, the researcher conducted peer debriefing with the supervisors in Malaysia throughout the research. It provides the researcher with an objective perspective of the quality and content of data collection. The researcher shared the data transcripts with the supervisors.

### 2.7 Ethical approval

This study was approved by the Ethics Committee of Jingjiang People’s Hospital (KY 2022–167-01), and adhered to the principles of the Declaration of Helsinki. All participants were informed of the purpose and procedures of the study, and written informed consent was obtained from them before the interviews. The trial was registered in November 2022 at Clinical Trials.gov (NCT05606549).

## 3 Results

A total of 18 couples were invited to the study, and 18 couples were qualified. There were no withdrawals during the interview process. Among the 18 patients, 5 (27.78%) were females, and six (33.33%) were aged between 50 and 60. Five (27.78%) patients fell within the 60–70 age bracket, six (33.33%) were aged between 50 and 60 years, six (33.33%) were aged >70 years, one (5.56%) were between 20 and 30 years. The spouses spanned several age groups: one (5.56%) was between 31 and 40 years, one (5.56%) was between 41 and 51 years, five (27.78%) were between 51 and 60 years, and six (33.33%) were between 60 and 70 years, five (27.78%) were aged >70 years. Nine patients (50.0%) had undergone gastric surgery. The patients’ and their spouses’ educational backgrounds ranged from elementary to undergraduate, with half of the patients and spouses having attended middle school (9 cases). Eight (44.44%) couples reside in rural areas, and seven (38.89%) live in urban areas, three (16.67%) live in urban areas. Among them, six couples (33.33%) have a per capita monthly household income below 2000 Yuan, six couples (33.33%) have a per capita monthly household income ranging from 2001 to 4,000 Yuan, while three couples (16.67%) have incomes ranging from 4,001 to 6,000 Yuan, and two couples (11.11%) have incomes ranging from 8,001 to 10,000 Yuan. Detailed information about the participants is available in Table 2.

**Table 2:** General information of participants.

Patient	Gender	Age (years)	Sessions Length of Marriage	Residence	Per capita monthly household income(Yuan)	Educational level	History of surgery	Cancer staging	Spouse	Age (years)	Educational level
P1	Male	61–70	>30	Rural	2001–400	Elementary School	Y	III	S1	61–70	Elementary School
P2	Female	61–70	>30	Town	2001–4,000	Elementary School	N	III	S2	61–70	Middle School
P3	Male	61–70	21~30	Urban	4,001–6,000	Elementary School	Y	II	S3	61–70	High School
P4	Female	51–60	21~30	Rural	<2000	Elementary School	N	IV	S4	51–60	Middle School
P5	Male	>70	>30	Rural	<2000	Elementary School	N	III	S5	>70	Elementary School
P6	Male	51–60	21~30	Urban	2001–4,000	Middle School	N	III	S6	51–60	Middle School
P7	Male	>70	>30	Town	<2000	High School	Y	III	S7	>70	Middle School
P8	Female	>70	>30	Rural	6,001–8,000	Middle School	N	IV	S8	>70	Elementary School
P9	Male	61–70	>30	Rural	<2000	Elementary School	Y	III	S9	61–70	Elementary School
P10	Male	>70	>30	Urban	6,001–8,000	Middle School	Y	III	S10	>70	Elementary School
P11	Male	>70	>30	Urban	6,001–8,000	Middle School	Y	IV	S11	>70	Middle School
P12	Female	51–60	21~30	Rural	4,001–6,000	Middle School	Y	IV	S12	51–60	Middle School
P13	Male	51–60	21~30	Rural	8,001–10,000	Middle School	Y	III	S13	41–50	High School
P14	Female	20–30	<10	Urban	8,001–10,000	Undergraduate	N	II	P14	31–40	Undergraduate
P15	Male	51–60	21~30	Urban	<2000	High School	N	II	S15	51–60	High School
P16	Male	>70	21~30	Rural	2001–4,000	Middle School	N	IV	S16	61–70	Middle School
P17	Male	51–60	21~30	Town	4,001–6,000	Middle School	Y	IV	S17	51–60	Middle School
P18	Male	61–70	>30	Urban	<2000	Middle School	N	IV	S18	61–70	Middle School

This study employs traditional content analysis for data analysis, revealing five overarching themes and 15 sub-themes derived from the experiences of patients with gastric cancer and their spouses during chemotherapy. The framework of the marital self-disclosure experience is shown in Table 3.

**Table 3:** Theme and subtheme.

Theme	Subtheme
Perception of marital self-disclosure	The Necessity of Marital Self-disclosure
	The Importance of Gradual Marital Self-disclosure Among Couples
The Influence of marital self-disclosure on Couples’ Well-Being	The Role of marital self-disclosure in Alleviating fear of cancer recurrence
	Enhancing Intimacy and Improving Coping Abilities
	Balancing Psychological Adaptation
Addressing Concerns about marital self-disclosure	Fear of Burdening the Partner
	Communication Challenges
	Shifting Dynamics in Family Roles
Factors Facilitating marital self-disclosure	Effective Communication Skills
	The Spouse’s Trust and Support
	Treatment benefit
	Communication Environment
The Obstacles of marital self-disclosure	Negative Communication
	Illness related changes
	Negative Emotional Interference

### 3.1 Perception of marital self-disclosure

In interviews with 18 patients with gastric cancer and their spouses, the understanding of self-disclosure among couples was primarily reflected in two aspects: the necessity of self-disclosure among couples and the recommendation that self-disclosure among couples should be approached “gradually.”

#### 3.1.1 The necessity of marital self-disclosure

With the gradual integration of positive psychology concepts in clinical settings, patients perceive marital self-disclosure as essential. It aids in uncovering each partner’s challenges and alleviating illness-related stress, fostering hope, and encouraging open communication in their daily interactions.

“*I actively communicate with my husband, and he can also proactively detect my difficulties and suffering. I think self-disclosure among couples is very important*.” (P14).

“*For me, it’s really important. We have good communication; we talk about everything, measuredly and timely. I tend to talk a lot, I always express my thoughts, and then we often lighten the mood together, telling jokes. Talking about it is a great way to release pressure, as long as you are open-minded. He keeps me optimistic, not shying away from issues, and we address problems promptly*.” (P15).

“*Looking at it now, disclosure is very important. After talking, our relationship has improved, expressing ourselves makes us feel more comfortable inside, hopes are higher, and so is confidence*.” (P17).

#### 3.1.2 The importance of gradual marital self-disclosure among couples

Patients undergoing chemotherapy for gastric cancer often endure a decline in their physiological condition, manifesting in various digestive symptoms. Initially, their concerns revolve around the disease itself, but over time, they broaden to include social interactions and the exploration of potential benefits and prospects. Some participants believe gradual self-disclosure is necessary.

“*These four nurse-led sessions covered different directions of communication, which I found to be very reasonable. At first, I did not want to say much because the burden of the disease weighed heavily on me. But after gradually coming to terms with it, I felt much better and started to share positive things with my spouse, like future plans*.” (P3).

“*In the beginning, our conversations were only about the disease. Later on, as my thoughts became more open, I could talk about many positive things. Such arrangements are really good; if we had started with other topics (social interactions, discovering benefits, future prospects), I would not have been able to say much. The guidance from the nurses was very good. Listening to the nurses and following through with self-disclosure, persistence is victory*.” (P6).

### 3.2 The influence of marital self-disclosure on couples’ well-being

Following a 16-week intervention centered on spousal self-disclosure, participants overwhelmingly reported positive outcomes, notably evident in three key areas: reduced fear of cancer recurrence among spouses, enhanced coping abilities, and improved psychological adaptation.

#### 3.2.1 The role of marital self-disclosure in alleviating fear of cancer recurrence

Marital self-disclosure guided both couples to express their concerns about cancer recurrence or progression, identify specific fears, and provide relief. Its objectives included challenging erroneous beliefs, promoting the expression of positive emotions, recognizing beneficial aspects, and assisting patients in envisioning and planning for the future. Creating a supportive environment where patients could voice uncertainties facilitated discussions about the fear of recurrence, ultimately leading to its reduction among couples.

“*Self-disclosure (between spouses) can increase the confidence to overcome the illness. My wife and I often talk about the good aspects and actively find joyous things. Gradually disclosing these feelings has made us less afraid (of the disease recurring).”* (P1).

“*Through our communication over this period, my partner has helped alleviate my fears. Now, I am not so afraid of the illness anymore. The last time I coughed up blood, they were very worried, but I told them not to worry, that I did not have any major issues and did not feel anything. Now, I’m not worried anymore, and I feel that I am recovering quite* well.” (P2).

“*Through mutual disclosure with my partner, I have slowly come to accept and not be afraid. Communicating more improves one’s mood. Although I feel that medication and chemotherapy can be harmful, after disclosing these feelings to him and receiving his constant reassurance, I now feel much less burdened.”* (P4).

#### 3.2.2 Enhancing intimacy and improving coping abilities

Active communication often results in an increase in intimacy and mutual understanding among patients. The positive attitude displayed by their spouse frequently serves as a counterbalance to their own negative emotions.

“*After I shared my health concerns with her, she immediately made me a ‘Five-Red Soup’ to replenish my energy. Aside from providing substantial help in my daily life, my old lady (wife) has also greatly supported my mental well-being. We’ve become closer.”* (P3).

“*We talk much more than before. Now that we communicate more, it has greatly helped to strengthen our bond and enhance our mutual understanding. Just talking things out can really brighten our mood.*” (P12).

#### 3.2.3 Balancing psychological adaptation

Self-disclosure interventions, akin to positive cognitive process therapy, play a crucial role in helping patients reevaluate and adapt to stressful situations, ultimately alleviating psychological distress. Initially, some patients may harbor a negative attitude toward treatment, particularly when faced with the pronounced side effects of chemotherapy. However, through ongoing marital self-disclosure, patients’ psychological adaptation gradually improves, leading to a notable reduction in stress and psychological burden.

*“After opening up, both our attitudes have improved. Just live for today without overthinking or worrying too much.”* (S7).

*“I talk to her about my stress, and just talking about it somehow makes it seem less significant. Now, I feel less burdened, less stressed, happier, and not as anxious.”* (P11).

*“My spouse always comforts me, saying that not getting worse is progress. The disease, cancer, being under control signifies improvement. So, I feel much better now.”* (P12).

### 3.3 Addressing concerns about marital self-disclosure

Patients with gastric cancer undergoing chemotherapy frequently contend with a range of discomforts. However, they often hesitate to share their burdens with their partners for fear of burdening the partner psychologically or meeting communication challenges (especially the male patients), and fear of challenging their autonomy in decision-making, potentially leading to excessive protection.

#### 3.3.1 Fear of burdening the partner

Following diagnosis, some patients may perceive themselves as unable to contribute to their family and fear becoming a burden to their loved ones. Consequently, they may opt to suffer in silence, avoiding communication out of concern that expressing their negative emotions would only add psychological burdens and trouble to their spouses.

*“He’s already having a tough time; I’m actually very worried about him. Sharing some things, I’m afraid it would make him uncomfortable.”* (P12).

*“Sometimes, I do not feel like talking, I feel terribly bad, I do not want to talk or say too much, afraid it would make her worried, afraid it would stress her out.”* (P6).

*“Sometimes, when I see him particularly busy, I also get anxious, but I cannot help, I can only digest the stress myself, not wanting to trouble him more.”* (P11).

#### 3.3.2 Communication challenges

Certain male patients, who are not adept at verbal communication, may rely on non-verbal cues rather than direct verbal expression in their marriages. They may feel embarrassed to articulate their thoughts verbally, often appearing “tongue-tied,” and may struggle with expressing fear or gratitude due to feelings of shyness.

*“I’m rough inside, cannot express many things, many mushy, thankful words I cannot say.”* (P3).

*“At the beginning, I was too embarrassed to say anything. Even now, sometimes, I still feel embarrassed; expressing it seems too mushy. I am from county, more down-to-earth, not good at saying nice things.”* (P17).

#### 3.3.3 Shifting dynamics in family roles

The spouse assumes responsibility for managing the patient’s medication, monitoring symptoms, scheduling treatments, and handling daily household tasks. As a result, the patient may relinquish their previous role as the “head of the family” or the primary household manager. This shift can occasionally lead to conflicts, particularly if the spouse becomes overly protective.

*“I am very proactive; I arrange everything at home. I am very initiative. I have everything in life arranged perfectly. I fear that after showing my vulnerability, I will become the one who is pitied, then, she manages our whole family life.”* (P3).

*“I am often very scared of undergoing examinations, always having CT scans. I am afraid that after revealing my fear, she keeps pushing me to go for the examinations.”* (P15).

### 3.4 Factors facilitating marital self-disclosure

Effective communication skills, mutual understanding, and trust between spouses, along with successful disease treatment, a supportive and familiar communication environment, and the spouse’s attentive care, all contribute to promoting self-disclosure within couples.

#### 3.4.1 Effective communication skills

The nursing staff offers comprehensive guidance on self-disclosure for both patients and their spouses, facilitating the patients’ engagement in this process. Through affirmation and appropriate encouragement provided during follow-up visits, nurses often boost patients’ confidence, thereby fostering increased self-disclosure within the couple.

*“After getting sick, the communication methods guided by the nursing staff changed my temper a lot. I do not lose my temper anymore and can communicate calmly. When urgent matters arise, I do not let them linger overnight but provide timely feedback.”* (P1).

*“Whoever gets this disease knows it best. At first, I would not talk, and I was gloomy every day, worrying inside. After some training over this period, I’ve started to speak out about my worries.”* (P4).

#### 3.4.2 The spouse’s trust and support

Patients have highlighted the significance of their spouse’s understanding and trust as positive reinforcement for self-disclosure. Feeling secure in the knowledge that they will not face criticism and that their vulnerability was been met with understanding encourages patients to open up. As emotional support increases, intimacy deepens, and emotional communication improves, fostering a cycle of ongoing self-disclosure between spouses.

*“I express my gratitude to her and thank her for taking good care of the home. I share all my worries with her. She understands me and truly wants what’s best for me, taking good care of me every day, not letting me do anything at home, and she never complains.”* (P1).

*“After I opened up to him, he understood me, which made me happy, and I wanted to share more with him.”* (S7).

*“I am also very thankful for his trust in me. When she talks, I am very willing to listen.”* (P10).

During chemotherapy, patients with gastric cancer experience a decline in physiological function and are partially limited in activities, leading to a decrease in their ability to care for themselves. They frequently rely on the attentive care of their spouses to meet their daily needs. During this period, understanding and caring for the patient by the spouse can offer significant physical and psychological support, boosting the patient’s confidence in battling the disease and encouraging their self-expression.

*“In the morning when I come to the hospital, he never leaves my side, taking care of me meticulously. My husband treats me very well; his service is spot-on. He watches me eat. Every time I go out, he calls me, asking me to come home and eat. My recovery is all thanks to the good care of my spouse. I find it easier to let go of my guard and talk to him about the illness, about my worries.”* (P2).

*“Everything else is fine. My wife arranges my life exceptionally well, and I am willing to disclosure to her.”* (P10).

#### 3.4.3 Treatment benefit

As treatment progresses, the patient’s clinical symptoms gradually diminish, and their confidence in recovery grows. Through a deeper understanding of the illness, patients develop a more positive outlook, which aids in emotion regulation, mitigates negative feelings, and improves psychological well-being. Consequently, this enhanced mindset promotes better adherence to conduct marital self-disclosure.

*“His mentality is also very good. People who know him say, ‘Did the doctor make a mistake? He does not seem sick at all’ He also maintains a cheerful mood and mental health. In fact, we just believe in the doctor’s ability. With a good attitude, you are willing to say anything.”* (S2).

*“Now I sleep much better, the disease is under control, and my mindset has improved. I feel better every day, so I’m more willing to express myself to her.”* (P6).

#### 3.4.4 Communication environment

Numerous participating couples have noted that in a relaxed environment, particularly when they are alone at home, expressing themselves becomes more comfortable. Some couples even incorporate self-disclosure sessions into their daily routine, finding that such an environment feels relatively “safe” and has minimal impact on the patient’s negative psychological state. Engaging in individual recreational activities, such as playing cards, helps them unwind, alleviate anxiety, and foster discussions on sensitive topics.

*“When we are at home, just the two of us, the environment for expression is more relaxed. We say whatever comes to mind, share everything, and talk about any discomfort.”* (P6).

*“Our daily routine is to have dinner at 6, take a walk at 8, have dinner at 10, do some Tai Chi, take a shower, soak in the bath, relax and talk to my partner, share my happiness of the day. Sometimes, she accompanies me to play cards to relax, and we talk (express).”* (P9).

### 3.5 The obstacles of marital self-disclosure

The obstacles of marital self-disclosure are relative to the factors that promote it. Participants mentioned impediment factors such as “conflict communication, changes in illness, negative emotional interference,” which can hinder the conduct of marital self-disclosure.

#### 3.5.1 Negative expression of emotions

After falling ill, some patients experience certain physiological pain. Coupled with negative events in life, some patients cannot control their temper, exhibiting silence, irritability, and outbursts, resorting to negative communication as a way to vent their psychological distress.

*“I cannot control my temper. In fact, I do not quarrel with her, but she gets angry, in fact, I get good after I lose my temper.”* (P5).

*“I went in at twelve and did not come out until one o’clock. I was so anxious, so I argued with her.”* (P11).

Spouses also stop communicating with negative emotions to avoid conflict communication.

*“Sometimes communication leads to quarrels, sometimes I get angry. If I do not want to confront him, I will not say anything.”* (S7).

*“Sometimes it’s easy to quarrel when disclosing. I can only give in to him and not say anything for now.”* (S1).

#### 3.5.2 Illness related changes

As the illness progresses, patients often face repeated hospitalizations, resulting in significant treatment expenses and uncertainty regarding the effectiveness of chemotherapy. This uncertainty can trigger anticipatory grief, leading to feelings of fear, self-blame, and helplessness. Heightened sensitivity to bodily symptoms further undermines the patient’s confidence in managing the disease and contributes to negative emotions like anxiety and depression. Consequently, patients may become reluctant to disclose their feelings in such challenging circumstances.

*“A few days ago, my platelets were low, and I did not feel like talking.”* (P8).

*“After chemotherapy and returning home from check-ups, I feel burdened. Then, when it comes to disclosure, I do not want to say anything. At that time, I’m afraid my husband will comfort me because my mood improves only superficially; I’m still worried inside, so I do not want to talk.”* (P4).

#### 3.5.3 Negative emotional interference

Patients may experience low moods or even depression due to the influence of adverse events, resulting in blank thoughts and unwillingness to share or disclose.

*“When I was waiting for a bed, there were a lot of patients. Sometimes, it’s been a few days late, and I could not get a bed in the morning or afternoon. I do not want to go to the hospital anymore, feeling bad and not wanting to share anything with her.”* (P1).

*“Sometimes I worry and feel bad, feeling that the pain has no end, not knowing when it will end. I do not know how to express it.”* (P12).

## 4 Discussion

### 4.1 Patients with gastric cancer and their spouses believe it is necessary to engage in marital self-disclosure, and gradual disclosure leads to better outcomes

This study found that patients with gastric cancer and their spouses consider marital self-disclosure necessary. Self-disclosure influences interpersonal relationships and promotes the development of such relationships (Zhang et al., 2019). Through self-disclosure, both parties interact, understand, penetrate, and exchange information, and intimate relationships develop into ones that are more conducive to maintaining and consolidating marital relationships. Therefore, the participants of this study all affirmed the necessity of marital self-disclosure, and also stated that self-disclosure can facilitate one party in discovering the difficulties and pains of the other, alleviate the release of disease pressure, and be willing to engage in marital self-disclosure actively in daily life.

The marital self-disclosure intervention program constructed in this study is gradual, progressing from Personal Emotional Expression, Social Cognition Expression, Benefit Discovery, and Outlook to The Future, sequentially, in line with the psychological changes of cancer patients from shock and denial to acceptance. Initially, attention is focused on Personal Emotional Expression and Social Cognition Expression, prompting patients to focus on their emotional needs, encouraging couples to express their disease experiences and feelings, requiring other parties to listen and provide timely feedback, enhancing communication and active coping between couples. In the third and fourth phases, couples are gradually guided to discover benefits and plan for the future, using positive psychology methods to promote positive emotions in patients and their spouses, positively facing future life, and enhancing the joint coping ability of patients and spouses. Research (Qianqian, 2016) has found that positive self-disclosure between patients and spouses not only improves intimacy between patients and spouses, reduces perceived caregiving pressure on spouses, promotes the psychological health of both parties but also enhances the quality of life of couples, promoting physical and mental health. However, some patients with gastric cancer also believe that self-disclosure needs to proceed slowly because chemotherapy leads to a decline in physical conditions, causing a series of symptoms in the digestive system. In the initial stage of treatment, patients focus more on the disease itself and have less desire for disclosure. This suggests that nursing staff must further explain the advantages of marital self-disclosure and the benefits of early intervention, enabling patients and their spouses to understand and implement it fully early on.

### 4.2 Mastering communication knowledge, a comfortable and familiar communication environment, and positive therapeutic effects are factors of marital self-disclosure

The study highlighted that mastering communication knowledge, creating a comfortable and familiar communication environment, and achieving positive therapeutic outcomes are facilitating factors for effective marital self-disclosure.

Research (Qiu, 2023) found that the level of communication knowledge directly predicts patients’ psychological distress and indirectly influences it through the mediating effect of intimate relationships. Patients’ understanding of marital self-disclosure knowledge affects their self-disclosure abilities, thereby impacting their level of engagement. This aligns with the findings of this study. Prior to each interview, patients and their spouses underwent self-disclosure method training in this study to enhance both parties’ communication skills, facilitate emotional expression, dissect genuine thoughts and feelings, aid in understanding how to engage in marital self-disclosure correctly, and underscore the importance of adequate disclosure and response by both listener and speaker. This process aims to foster a virtuous cycle, enhance both parties’ emotional regulation abilities, alleviate negative emotions, improve psychological states, and promote marital relationships. During training, nurses promptly corrected patients’ cognitive biases and maladaptive behaviors while providing affirmation and appropriate encouragement, thereby boosting patients’ treatment compliance and promoting spousal self-disclosure.

Positive treatment outcomes are influencing factors of patients’ positive emotions. The fewer clinical symptoms patients exhibit, the stronger their confidence in disease recovery becomes. Additionally, as patients deepen their understanding of the disease, they gradually develop a positive perception of it, which promotes positive feedback in disclosure and strengthens the desire to disclose. As patients’ disease treatment outcomes improve, they feel more relaxed, leading to a greater willingness to disclose (Qiu, 2023).

The research results (Ting, 2022) indicate that the quality of communication between spouses influences the patient’s enthusiasm in coping with cancer. Knowledge of communication and familiarity with the communication environment is essential for promoting self-disclosure between spouses. Nearly all couples mentioned in this study that communication in daily life is a recurring issue and the most frequent topic of exchange. Olson et al. (1983) argue that effective communication – involving exchange, listening, disclosure, clarity, respect, and active attention – can promote patient understanding of family and external changes while maintaining balance. Manne et al. (2014) emphasize that good self-disclosure enables spouses to face illness challenges cooperatively. Therefore, patients with gastric cancer and their spouses actively seek suitable and effective communication methods while at home. A comfortable and familiar communication environment can facilitate self-disclosure, making spouses feel relatively “safe” to express themselves daily. Patients and their spouses are more willing to discuss privacy issues and life challenges freely. Daily life topics, such as dietary needs and treatment-related requirements, are the simplest for positive communication. Choosing the right timing and communication techniques is crucial for fostering effective and constructive interaction between spouses. The correct timing and a comfortable environment make it easier for patients to accept and enhance positive communication outcomes. Activities like daily routines, walks, or engaging in the patient’s favorite leisure activities (playing cards) can help them relax, alleviate anxiety, and pave the way for discussing delicate topics.

### 4.3 Marital self-disclosure can enhance couples’ intimacy, coping ability, psychological adaptation, and reduce the fear of cancer recurrence for couples

The study uncovered that marital self-disclosure not only enhances the intimacy, coping abilities, and psychological adaptability of couples but also reduces the fear of cancer recurrence for both spouses. The Relationship Resilience Model suggests mutual dependence between spouses can increase psychological resilience (Jiang Liling et al., 2018; Ting, 2022). Disease serves as a common stressor for both spouses and behaviors conducive to maintaining or enhancing marital intimacy can improve the psychological well-being of both parties (Jiang Liling et al., 2018; Ting, 2022). The Interpersonal Intimacy Process Model posits that establishing intimate relationships is a dynamic developmental process, with self-disclosure and the spouse’s response crucial (Müller et al., 2000; Parise and Caggiano, 2018). Research (Luo Qun et al., 2016; Porter et al., 2018) also suggests that good communication between spouses not only facilitates the establishment of solid marital intimacy but also improves the health outcomes of the spouses. This aligns with the findings of this study, which propose that spousal intimacy results from self-disclosure being a facilitating factor. Self-disclosure is considered necessary for forming and developing intimate relationships and is the most critical predictor of such relationships. Research (Ting, 2022) has found a close association between self-disclosure and psychological distress, with households with higher levels of self-disclosure and more excellent spousal communication exhibiting higher marital satisfaction (Lee et al., 2020; Qi et al., 2022). Effective self-disclosure can reduce the occurrence of anxiety and depression in spouses. This study found that self-disclosure can promote intimacy between spouses, deepen emotions, enhance confidence in disease treatment, and improve psychological well-being. This is similar to the findings of Luo Zhen et al. (2022), who discovered that primarily wife-initiated self-disclosure interventions can alleviate the psychological distress of prostate cancer radiotherapy patients and enhance marital intimacy satisfaction.

The participants in this study also indicated that the positive dyadic coping strategies between patients and spouses not only improve the intimacy between them but also alleviate the perceived caregiving stress for spouses and promote the psychological well-being of both parties. Research (Zhaoyang et al., 2018) has demonstrated that open communication between spouses significantly impacts the dyadic coping of patient-spouse pairs, enhancing their proactive coping abilities. This study also found that the understanding and trust of spouses and their attentive care can promote marital self-disclosure. Some patients mentioned that the understanding and trust of their spouses constitute positive feedback for disclosure, making patients feel that disclosure is safe, non-judgmental, and vulnerabilities are understood. With more emotional support, higher intimacy, and better emotional communication, the continuous engagement of marital self-disclosure is facilitated. During chemotherapy for patients with gastric cancer, their physiological functions decline, and some activities are restricted, leading to a decrease in self-care ability. They often require the care and assistance of their spouses to meet daily needs. At this time, the understanding and attentive care of spouses can greatly comfort patients, strengthen their confidence in fighting the disease, and thus promote disclosure. Positive self-disclosure, in turn, can promote couples’ intimacy, coping abilities, and psychological adaptability. This study guides both spouses to express their concerns about cancer recurrence or progression, identify specific fears, and alleviate the fear of cancer recurrence through communication. It aims to change erroneous beliefs, encourage the expression of positive emotions, identify beneficial aspects, and guide patients to anticipate and plan for the future. It allows patients to express uncertainty in a supportive environment and facilitates discussions about the fear of recurrence, enabling couples to face and gradually overcome it.

### 4.4 The partner unwillingness to trouble the other part, difficulty expressing oneself, loss of initiative, etc., are concerns about marital self-disclosure

Challenges in marital self-disclosure include the fear of the partner’s psychological unacceptance, reluctance to burden the partner, difficulty in expressing thoughts, inability to initiate conversation, and concerns about losing autonomy. Protective concealment within marriage implies that spouses or patients usually avoid communicating about sensitive topics to prevent causing worries or negative emotions in the other party, protecting the spouse from harm (Lupinacci et al., 2022; Moreira et al., 2010; Yu and Sherman, 2015) has found that both cancer patients and their spouses engage in protective concealment. In order to alleviate the burden on the patient or spouse, when they perceive that the diagnosis and treatment would cause harm to the spouse, they conceal their concerns; to avoid emotional harm, patients and spouses may choose to avoid communicating about the illness. Some couples may have good communication on non-cancer-related topics but insufficient communication on cancer-related issues, accompanied by communication pressure related to cancer. Generally, cancer-related communication may be related to marital relationships, with patients and partners more inclined to sacrifice cancer-related emotional support to maintain balance (Liu Zhiwei et al., 2023).

Some participants encountered negative emotions throughout the illness and chemotherapy process, including stress, sadness, fear, and depression. Patients often chose to endure silently, avoiding communication, fearing that expressing these negative emotions would further distress their spouses. These findings are consistent with those of Cheng et al. (2021). Uncertainty among spouses about the prognosis of gastric cancer led to a decrease in communication awareness and enthusiasm. Therefore, patients and their spouses sometimes have concerns about marital satisfaction and hold pessimistic views about their future (Liu et al., 2022). This study demonstrates that spouses who typically dominate in family communication are less inclined to show vulnerability or express their hardships proactively. This reluctance may stem from their historical roles as “head of the household” or “dominant lady.” However, these roles change with the progression of the disease, placing them at a disadvantage. These changes lead to emotional turmoil, making them more likely to avoid issues and withhold emotions. These insights are consistent with the findings of Yu and Sherman (2015).

It is noteworthy that there are certain gender differences in the marital self-disclosure. Male patients tend to exhibit more restraint and emotional suppression during marital self-disclosure, often opting for “silence” or simplified expressions in an effort to maintain family stability and avoid adding psychological burden to their spouses. In contrast, female spouses are more inclined to actively express emotions and concerns, aiming to enhance emotional connection and mutual support (Lu and Stanton, 2010). This phenomenon is closely related to traditional gender role expectations in Chinese culture, where men are typically expected to be strong and emotionally reserved, while women often take on a more active role in emotional communication (Lu and Stanton, 2010). Additionally, female patients are more likely to seek emotional responses and understanding when expressing physical or emotional distress, whereas male spouses may sometimes avoid or rationalize these situations due to a lack of experience in managing emotional expression (Lu and Stanton, 2010). These gender differences can influence the emotional responsiveness and the development of intimacy between couples. Future research could focus on the internal experiences of one gender (male or female), and intervention designs may consider a gender perspective to provide more targeted communication and psychological support strategies for both patients and their spouses.

### 4.5 Negative communication, changes in illness, and interference from negative emotions are obstacles to marital self-disclosure

The study highlighted that negative communication, changes in health conditions, and emotional disturbances as key obstacles to marital self-disclosure. During chemotherapy, patients with gastric cancer grapple with pronounced physical symptoms and psychological stress stemming from the adverse effects of the drugs. If these negative emotions remain unaddressed over a prolonged span, patients might display heightened irritability, anger, and emotional eruptions, using aggressive communication as a release. Such detrimental communication erodes the trust between patients and their spouses, nudging them toward more negative communication tactics, such as conflict-driven communication, to safeguard their emotions (Shin et al., 2016).

Additionally, some patients indicate that as the disease progresses, they require repeated hospital admissions, incurring high treatment costs. They develop uncertainty about the efficacy of chemotherapy, are prone to anticipatory grief, and feelings of fear, self-blame, and helplessness. Simultaneously, their sensitivity to bodily symptoms increases, intensifying worries and fears about disease progression or recurrence, exacerbating the generation of negative emotions and hindering patients from engaging in marital self-disclosure.

This study discovered that some participants and their spouses encountered negative emotions, including stress, sadness, fear, and depression, throughout the illness and chemotherapy. Patients often opted to endure silently and evade communication, worried that conveying these negative emotions would strain their spouses more. These observations align with the findings from a study by Cheng et al. (2021). Spousal uncertainty concerning the prognosis of gastric cancer led to diminished communication awareness and enthusiasm. As a result, patients overlooked the significance of communication and held pessimistic views about their future (Liu et al., 2022). The barriers to emotional interaction and patience in the exchange between spouses can also be attributed to their limited awareness of disease-related communication.

The treatment and recovery process for patients with gastric cancer are long-term, during which spouses often bear the primary caregiving responsibility (Johansen et al., 2018; Saimaldaher and Wazqar, 2020) indicate that spouses not only need to be involved in various aspects of cancer treatment but also encounter risks associated with the patient’s symptom changes. They also need to meet the patient’s various needs and provide social, emotional, and economic support. Spouses report experiencing pressures and burdens from various aspects, including physiological, psychological, and social, when their caregiving abilities cannot meet the demands of the patient role (Grunfeld et al., 2004).

A significant characteristic of Asian countries is the strong family bond, where spousal caregiving is often seen as an obligatory act driven by moral duty, reciprocity, and religious obligations. Compared to Western cultures, which emphasize “open communication,” Chinese people prefer “sharing joys without worrying.” Their communication style is often more indirect, possibly influenced by Confucianism, where silence is considered a virtue. However, prolonged endurance can lead to psychological distress (Majestic and Eddington, 2019) and social isolation (Hu et al., 2018) among couples, reducing their willingness to express themselves and even impacting their mental health (Vázquez et al., 2019). Studies (Borges et al., 2017; Saimaldaher and Wazqar, 2020) are consistent with the findings of Tolbert et al. (2018).

This study further indicates that factors such as knowledge of self-disclosure, understanding and trust from one’s spouse, effective medical treatment, a comfortable and familiar communication environment, and attentive spousal care can all facilitate marital self-disclosure. Therefore, in addition to emphasizing the act of disclosure itself, healthcare providers should also pay close attention to the quality of marital self-disclosure. Comprehensive training in self-disclosure, promotion of mutual understanding and trust, attentive care, and the creation of personalized and supportive disclosure environments can all positively enhance mutual communication.

Particular attention should be paid to negative communication patterns, especially those that emerge during conflicts or shifts in the course of the illness. Cancer is not only a physical disease; it also profoundly alters patients’ social identities and family roles. Many patients transition from being caregivers or economic providers to becoming care recipients, a change that may provoke feelings of loss of control and helplessness, which in turn can diminish both the willingness and depth of self-disclosure. This is especially true for gastric cancer patients who are often forced to withdraw from household responsibilities due to physical decline during treatment, resulting in a sense of “role loss” and emotional distress.

Therefore, nursing interventions should aim not only to help patients accept these changes but also to adapt to them. At the same time, spouses should be guided to understand the implications and emotional impact of such role transformations in order to foster more empathetic communication and support. Nurses should focus on personalized communication strategies, promptly identify disclosure barriers, and provide tailored interventions. Additionally, integrating case management into patient care may help refine communication approaches and support more effective, individualized psychosocial interventions.

### 4.6 Limitations of this study

Due to the demographic profile of individuals affected by gastric cancer, most of the couples included in this study had a marriage duration of over 20 years, and the majority of patients were male. This demographic skew may have introduced some bias to the study results. During the interview, there were instances of disagreement between spouses, manifested by their responses being categorized differently for the same interview question. Consistent with previous research (Hodges et al., 2005; Zachariae et al., 2003), it is anticipated that discrepancies in communication between patients and their partners are significant sources of distress, potentially more severe than the multitude of issues reported by each individual. While there is a significant correlation between discrepancies in viewpoints between patients and spouses and marital intimacy, no significant difference in distress was found between couples with higher versus lower levels of disagreement. Further research in this area is warranted in the future.

### 4.7 Implications of the study

Oncology nurses are capable of assessing psychological disorders in cancer patients and providing appropriate evidence-based supportive psychological care for these disorders. Moreover, patients have expressed a desire for nurses to be present and provide support during cancer treatment, and psychological support provided by nurses appear to be more readily accepted by cancer patients experiencing psychological difficulties (Baker-Glenn et al., 2011; Rustøen et al., 2009). Future recommendations for communication training for oncology nurses may include several key components. First, emotional sensitivity training can help nurses recognize both verbal and nonverbal cues of distress in patients and their spouses. Second, training should address how to identify and manage gender-specific communication dynamics, such as emotional restraint or avoidance. Third, culturally sensitive communication skills are essential—particularly in the Chinese context, where concepts such as “face” and emotional control may influence information disclosure. The use of empathetic responding techniques—such as emotional validation, encouraging expression, and providing nonjudgmental support—can strengthen trust and emotional closeness between couples. Finally, role-playing and reflective practice can help nurses apply these skills in real-life scenarios, enhancing their confidence in addressing marital communication challenges. Integrating these components into nursing education and ongoing professional development can better equip oncology nurses to support couples jointly coping with the stresses of cancer. Cancer affects not only patients and their spouses but also the entire family. Changes in communication roles and family behavior dynamics can profoundly influence marital interaction. Future research could further explore family-level communication patterns among cancer patients.

## Data Availability

The raw data supporting the conclusions of this article will be made available by the authors, without undue reservation.
